# Supplementary material for: Promotion of Wound Healing and Prevention of Frostbite Injury in Rat Skin by Exopolysaccharide from the Arctic Marine Bacterium Polaribacter sp. SM1127
Source: Mar Drugs. 2020 Jan 11;18(1):48. doi: 10.3390/md18010048 (PMC7024241; doi:10.3390/md18010048)
Supplement: Supplementary file 1 [file marinedrugs-18-00048-s001.pdf]

Promotion of Wound Healing and Prevention of Frostbite Injury in Rat Skin by Exopolysaccharide from the Arctic Marine Bacterium *Polaribacter* sp. SM1127

Mei-Ling Sun [1], Fang Zhao[1], Xiu-Lan Chen [1,2], Xi-Ying Zhang [1,2], Yu-Zhong Zhang [1,2,3], Xiao-Yan Song [1], Cai-Yun Sun [1] and Jie Yang [1,*]

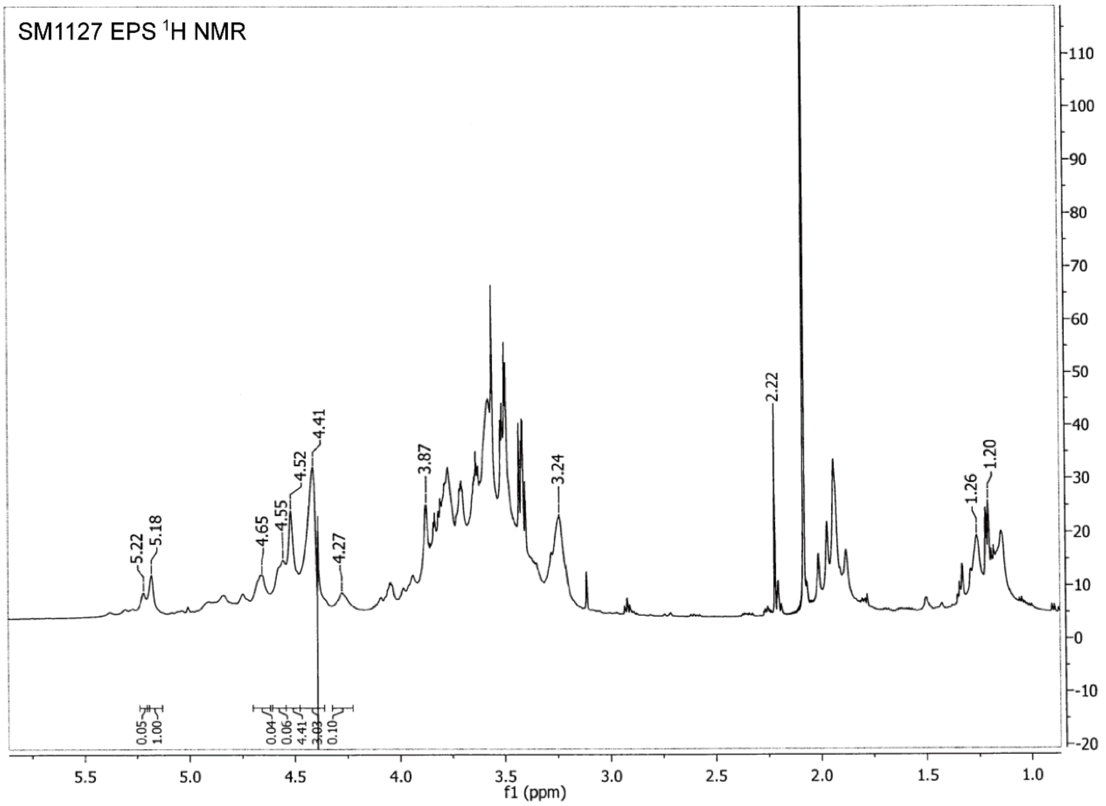

Figure S1. ^{1}H NMR spectrum of SM1127 EPS on a Varian Inova-500 MHz Bruker NMR spectrometer at 70°C (δ_H=2.225 ppm).

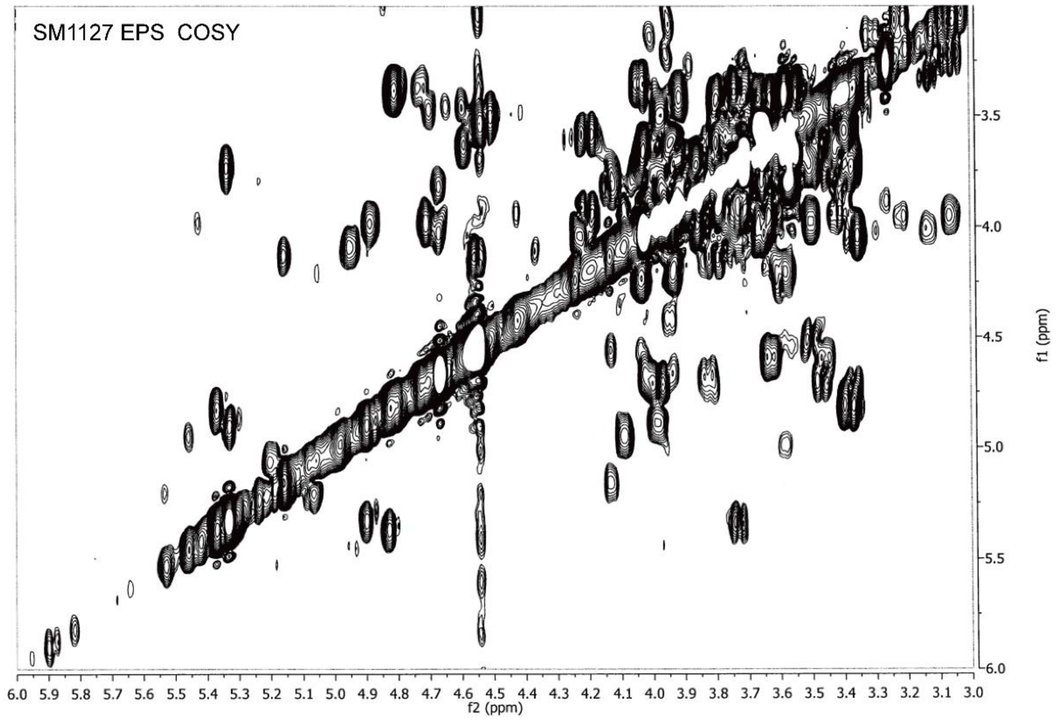

Figure S2. COSY spectrum of SM1127 EPS on a Varian Inova-500 MHz Bruker NMR spectrometer at 70°C (δ_C=30.89 ppm).

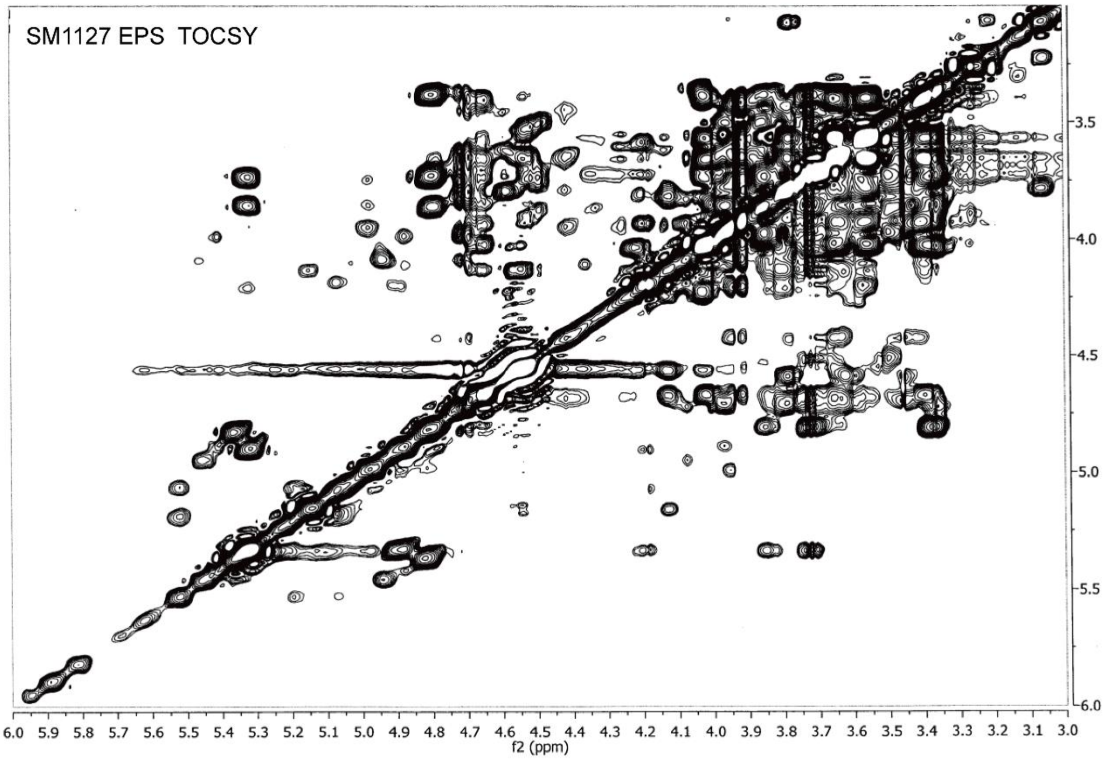

Figure S3. TOCSY spectrum of SM1127 EPS on a Varian Inova-500 MHz Bruker NMR spectrometer at 70°C (δ_C=30.89 ppm).

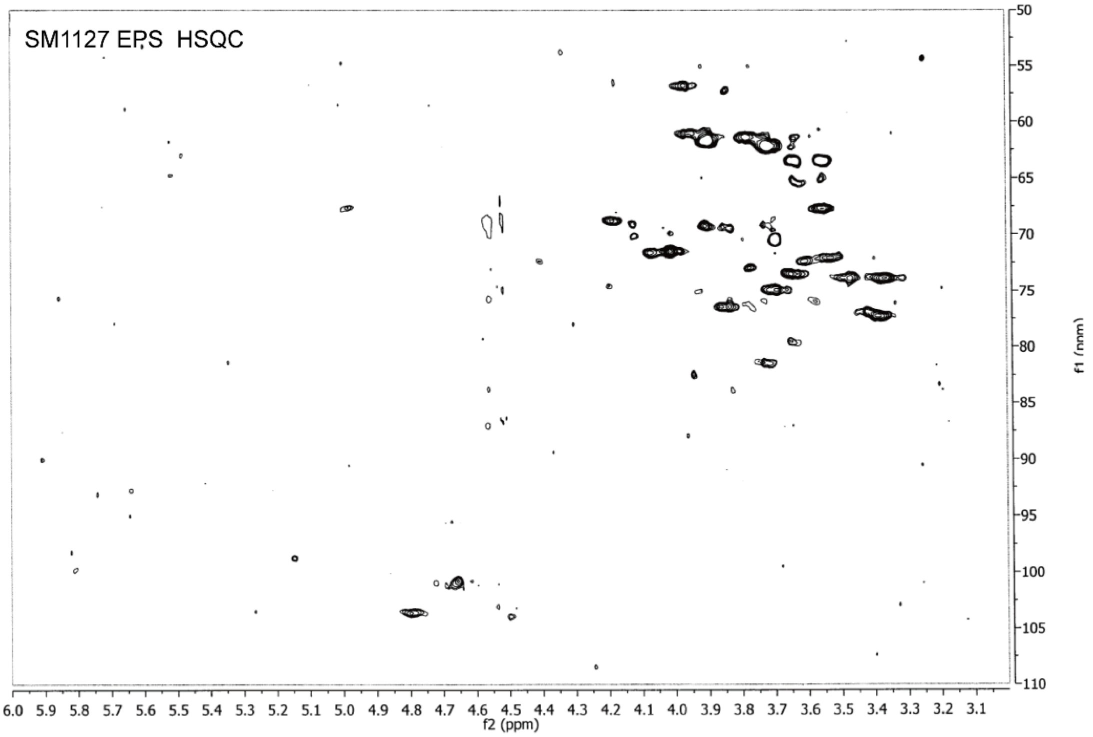

Figure S4. HSQC spectrum of SM1127 EPS on a Varian Inova-500 MHz Bruker NMR spectrometer at 70°C (δ_H=2.225 ppm, δ_C=30.89 ppm).
